# Novel Cationic Prodrug of Ubiquinol-10 Enhances Intestinal Absorption via Efficient Formation of Nanosized Mixed-Micelles with Bile Acid Anions

**DOI:** 10.3390/molecules25030546

**Published:** 2020-01-27

**Authors:** Shuichi Setoguchi, Ryoji Hidaka, Nami Nagata-Akaho, Daisuke Watase, Mitsuhisa Koga, Kazuhisa Matsunaga, Yoshiharu Karube, Jiro Takata

**Affiliations:** Faculty of Pharmaceutical Sciences, Fukuoka University, Nanakuma, Jonan-ku, Fukuoka 814-0180, Japan; ssetoguchi@fukuoka-u.ac.jp (S.S.); ryozy@orange.zero.jp (R.H.); nanohana_73@jewel.ocn.ne.jp (N.N.-A.); watase@fukuoka-u.ac.jp (D.W.); kogami@fukuoka-u.ac.jp (M.K.); karube@fukuoka-u.ac.jp (Y.K.)

**Keywords:** bioavailability, coenzyme Q_10_, drug delivery system, prodrug, ubiquinol, ubiquinone

## Abstract

The aim of this study was to develop a prodrug of ubiquinol-10 (UqH-10), the active form of ubiquinone-10 (Uq-10), for oral delivery. Bioavailability of UqH-10 is hampered by its high susceptibility to oxidation and water-insolubility. We prepared three novel *N,N*-dimethylglycine ester derivatives of UqH-10, including a 1-monoester (UqH-1-DMG), 4-monoester (UqH-4-DMG), and 1,4-bis-ester (UqH-DMG), and assessed their physicochemical properties in vitro and in vivo. UqH-DMG spontaneously formed an aqueous micelle solution comprising 20 nm particles at 36.5 °C. Cationic UqH-DMG formed nano-sized (5 nm) mixed-micelles with taurocholic acid. Reconversion of the derivatives to UqH-10 was accelerated in human liver microsomes. The oral bioavailability of UqH-10 after administration of UqH-derivatives or Uq-10 was determined in fasted and postprandial rats secreting normal and high levels of bile, respectively. In fasted rats, plasma UqH-10 after UqH-derivatives administration reached *C*_max_ at 2–3 h and after Uq-10 administration, it remained low. The *AUC*_0-24h_ of UqH-10 after UqH-derivatives administration was 2–3-fold higher than that after Uq-10 administration. In postprandial rats, the *T*_max_ of UqH-10 after UqH-derivatives administration was an hour earlier than after Uq-10 administration. In conclusion, cationic UqH-derivatives are convenient prodrugs that enhance UqH-10 bioavailability by forming nanosized mixed-micelles with intestinal bile acids.

## 1. Introduction

Ubiquinone-10 (Uq-10), better-known as Coenzyme Q_10_ (CoQ_10_), is widely distributed in mammalian tissues, membranes, and cells. Uq-10 is an essential component of the mitochondrial respiratory chain, where it acts as an electron carrier and participates in ATP production. Ubiquinol-10 (UqH-10), the fully reduced form of Uq-10, acts as an antioxidant within biological membranes and in the mitochondria by either scavenging free radicals directly or by recycling other antioxidants, like α-tocopherol and ascorbate [1,2]. Due to these beneficial roles, Uq-10 and UqH-10 are used to treat a variety of disorders, including cardiovascular syndromes and primary CoQ_10_-deficiency syndrome, and is available over the counter as a dietary supplement worldwide [3,4,5,6].

UqH-10 has powerful pharmacological activities, but its bioavailability is poor due to its low water-solubility (it is highly lipophilic) and its susceptibility to rapid oxidation. A way of improving the bioavailability of UqH-10 is by enhancing its diffusion efficiency in the unstirred water layer of the intestines, as this is a rate-limiting step in the intestinal absorption of lipophilic compounds [7]. Increasing aqueous solubility in the unstirred water layer is particularly important for efficient absorption of orally administered drugs. Several approaches have been tested to promote intestinal absorption, including formulating drugs in liposomes, nano-emulsions, or solid lipid nanoparticles. In particular, self-emulsified drug delivery systems (SEDDS) have been shown to induce bile secretion, leading to improved absorption [8,9,10,11,12]. However, these formulation techniques demand administration of large dosage volumes due to the requirement to include large amounts of fatty acids and surfactants. Smaller-volume formulations without excipients would make it easier to achieve target doses in recipients and for improved oral intake.

We hypothesized that a prodrug strategy for UqH-10 could overcome the aforementioned poor oral bioavailability. We previously reported that *N,N*-dimethylglycine esters of vitamin K_2_ hydroquinone, which, similar to UqH-10, contains phenolic hydroxyl groups and a large lipophilic side chain, had good water solubility [13] and improved bioavailability over the parent drug in rats [14].

**Figure 1:**
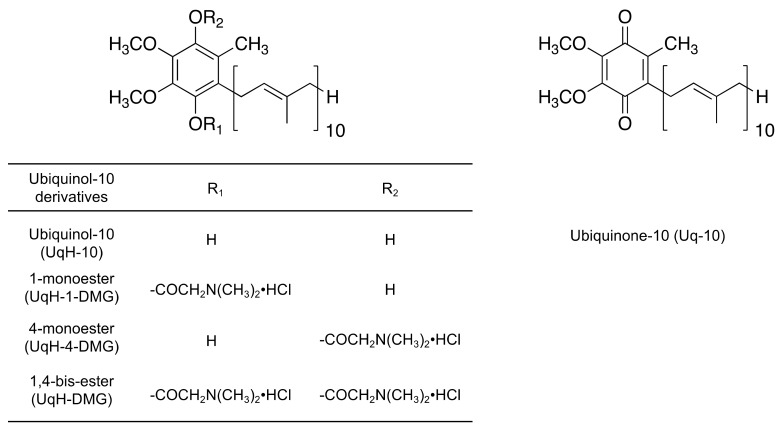
Chemical structures of Ubiquinol-10 derivatives and Ubiquinone-10.

The aims of the present study were to synthesize *N,N*-dimethylglycine esters of UqH-10 (Figure 1), evaluate their physicochemical properties in vitro, and their pharmacokinetics after administration as a UqH-10 prodrug in vivo. Since most previous studies aimed to induce bile secretion for the absorption of Uq-10 and UqH-10, we established an in vivo model where bile secretion in rats was regulated by controlled feeding to investigate the influence of bile secretion on the absorption of the UqH-10-derivatives. We provide evidence that UqH-10 *N,N*-dimethylglycine ester derivatives are prodrugs that can enhance intestinal absorption in small dosage volumes due to formation of mixed-micelles with endogenous bile acids.

## 2. Results and Discussion

### 2.1. Physicochemical Properties of UqH-10 Derivatives

Two types of monoester and a bis-ester were successfully obtained by chemical synthesis (Figure 1) and characterized by MS, elemental analysis, and ^1^H-^13^C HMBC NMR. The melting points of the monoesters (UqH-1-DMG, UqH-4-DMG) and the bis-ester (UqH-DMG) were approximately 80 °C and 180 °C, respectively (shown in Section 3.4). The melting points of these esters were much higher than that of Uq-10 (48 °C). UqH-DMG formed a transparent solution in water at concentrations up to 5 mM. Its water solubility was significantly higher than that of Uq-10, which was “practically insoluble, or insoluble” (even when diluted to < 0.116 mM) based on the Japanese Pharmacopoeia monograph. Both monoesters and the bis-ester were stable against oxidation under atmospheric conditions.

### 2.2. Micelle Formation of UqH-DMG in Water

**Figure 2:**
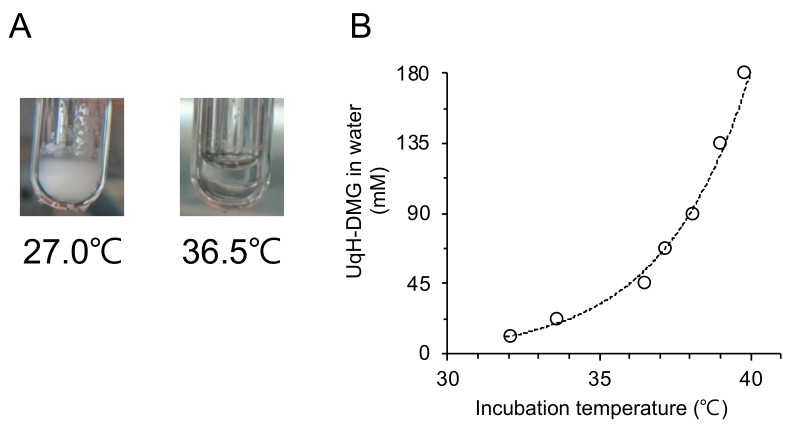
Influence of incubation temperatures on the solubility of UqH-DMG. (**A**) Representative photographs of 45 mM of UqH-DMG in water at 27.0 °C and 36.5 °C. (**B**) Solubilizing points of UqH-DMG in water versus incubation temperatures.

**Figure 3:**
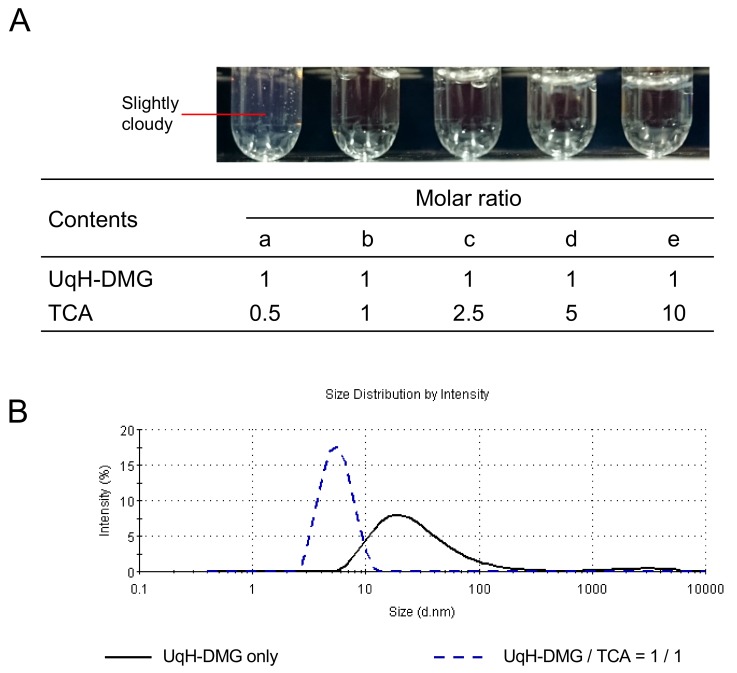
Effect of taurocholic acid on UqH-DMG solubility. (**A**) Appearance of mixtures of UqH-DMG and taurocholic acid (TCA) at different molar ratios. (**B**) Particle size distribution of UqH-DMG with or without TCA. Solid line: UqH-DMG only, dashed line: UqH-DMG:TCA at a molar ratio of 1:1.

To investigate the influence of temperature on the solubility of UqH-DMG, visual inspections of the solutions were performed in test tubes. A 45 mM of UqH-DMG dissolved in water appeared opaque at 27.0 °C, but after increasing the temperature to 36.5 °C, the solution became transparent, indicating that UqH-DMG formed a micelle solution (Figure 2A). The micelle size was confirmed in Section 2.3 below (Figure 3B). The solubilizing points at different concentrations of UqH-DMG were also measured and plotted (Figure 2B). An exponential increase in solubilizing point was observed immediately above 45 mM UqH-DMG, indicating that the Krafft’s point of UqH-DMG, a cationic large molecule, in water is around 36.5 °C. Therefore, it is likely that UqH-DMG in aqueous medium would be soluble at body temperature (~37 °C) after oral administration.

Water solubility of UqH monoesters (UqH-1-DMG and UqH-4-DMG) is lower than that of UqH bis-ester (UqH-DMG). Related Krafft’s points of the monoesters were not observed, since the effect of ionic surfactant of UqH monoesters were not observed due to the differences in the balance of polar group(s) and hydrophobic group between UqH bis-ester and monoesters.

### 2.3. Mixed-Micelle Formation of UqH-DMG with Taurocholic Acid

After UqH-DMG is solubilized in the stomach under low pH, it is expected that UqH-DMG will encounter bile acid anions in the intestinal lumen under neutral pH. Therefore, the interaction between UqH-DMG and taurocholic acid (TCA), a representative bile acid, was investigated. The appearance of a mixture of UqH-DMG:TCA at a molar ratio of 1: 0.5 (Figure 3A) was slightly cloudy, but at higher proportions of TCA, i.e., UqH-DMG:TCA ratios of 1:1 to 1:10, the solutions became transparent (Figure 3A). Solutions of 20 mM UqH-DMG in water and a mixture of UqH-DMG:TCA at a 1:1 molar ratio were subjected to dynamic light scattering measurements. Consistent with the appearances of the solutions shown in Figure 3A, the Z-average particle diameter of the 20 mM UqH-DMG solution was 20.38 ± 0.14 (mean ± SD) nm, higher than that of the UqH-DMG/TCA mixture, which was 5.183 ± 0.033 (mean ± SD) nm. These results suggest that the UqH-DMG: TCA mixture at a 1:0.5 molar ratio was cloudy because of complex formation, and at higher molar concentrations of TCA relative to UqH-DMG, mixed-micelles particles of approximately 5 nm in diameter form spontaneously. Small micelle particles under 10 nm in diameter are expected to diffuse efficiently in the unstirred water layer of the intestines, as this is a rate-limiting step in the intestinal absorption.

For the interactions between UqH monoesters and taurocholic acid, UqH-4-DMG was solubilized with, a 10-times molar-equivalents, taurocholic acid. A typical mixed-micelle is the state at which an anion-cation ion pair(s) is solubilized in either richer component. The types of micelle formed with taurocholic acid may differ between UqH bis-ester and monoesters.

### 2.4. Enzymatic Hydrolysis of UqH-10 Derivatives

To determine whether UqH-derivatives can be activated by liver esterases to revert to their parent form (UqH-10), their hydrolysis in rat and human liver microsomes was assessed. The UqH-DMG (bis-ester) is expected to be hydrolyzed in a sequential reaction via either 1-mono or 4-monoester, as previously reported for vitamin K hydroquinone [13]. Hence, the hydrolysis velocities of UqH-monoesters were compared. The velocity versus concentration profile was fitted using the Michaelis–Menten model, and the kinetic parameters were calculated using GraphPad Prism 6 (GraphPad Software, CA, USA) (Table 1). In rat liver microsomes, hydrolysis of UqH-4-DMG occurred much faster than UqH-1-DMG. The V_max_/K_m_ value of UqH-4-DMG was 8.9-fold higher than that of UqH-1-DMG. The hydrolysis of UqH-DMG was also assessed by the rate of generation of UqH-10. The K_m_ and V_max_ parameters were similar to those of UqH-1-DMG. Therefore, hydrolysis of UqH-DMG at position 1 is the rate-limiting step for conversion to UqH-10. In other words, the rates of hydrolysis depend on the esterified position on UqH-10. To simplify comparisons between experiments in rat and human microsomes, only UqH-4-DMG was subjected to a human microsome hydrolysis assay. UqH-4-DMG was readily cleaved in human liver microsomes, and the V_max_ and K_m_ values of UqH-4-DMG hydrolysis were similar to those observed in rat liver microsomes. Furthermore, to determine whether the hydrolysis of UqH-4-DMG was esterase-dependent, an esterase inhibitor, eserine, was added to the reactions. As shown in Supplemental Figure S1, eserine inhibited UqH-4-DMG hydrolysis in both rat and human liver microsomes in a concentration-dependent manner. These results strongly suggest that UqH-derivatives will be activated by the esterases in human livers.

### 2.5. Plasma Concentrations of UqH-10 after Single Oral Administration of UqH-Derivatives in Fasted and Postprandial Rats

**Figure 4:**
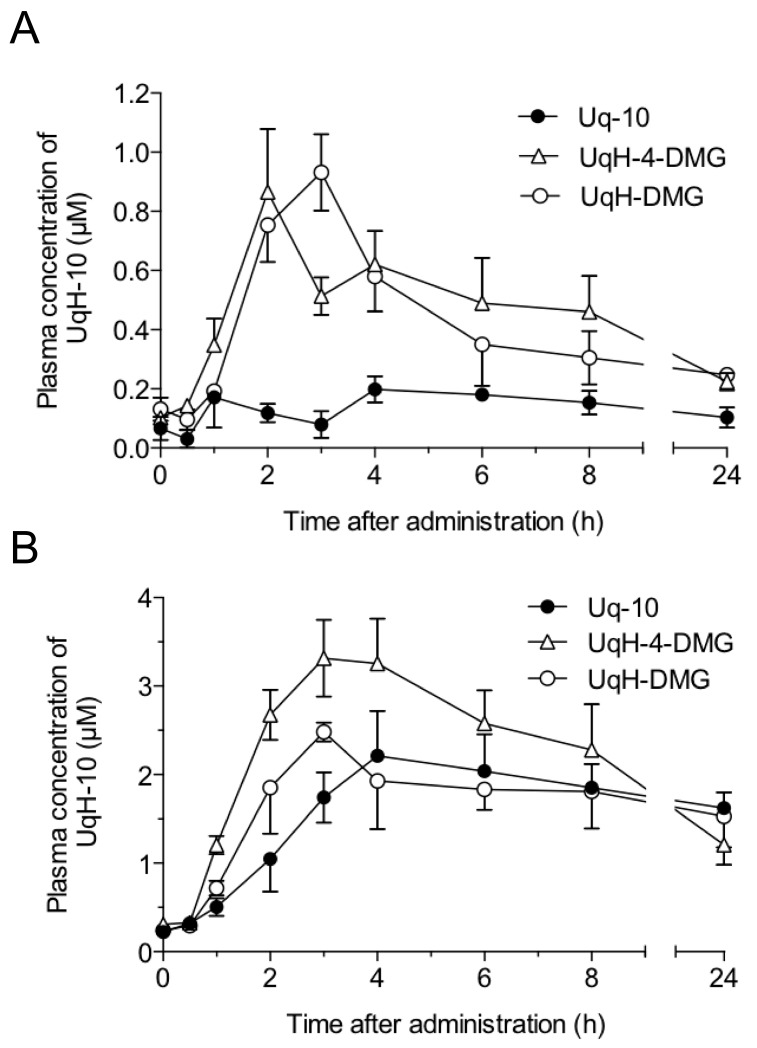
Plasma concentration of UqH-10 after administration of UqH-derivatives or Uq-10 in fasted and postprandial rats. (**A**) Plasma concentration of UqH-10 after administration of 40.5 mmol/kg weight of UqH-DMG, UqH-4-DMG or Uq-10 in fasted rats (UqH-DMG, n = 6; UqH-4-DMG, n = 5; Uq-10, n = 4); (**B**) plasma concentration of UqH-10 after administration of 40.5 mmol/kg weight of UqH-DMG, UqH-4-DMG or Uq-10 in postprandial state rats (n = 3). The values indicate mean ± SE.

To establish the utility of UqH-derivatives as prodrugs, we examined the plasma kinetics of UqH-10 after single oral administration of UqH-4-DMG and UqH-DMG in fasted rats. Based on preliminary tests, approximately 70% of total Uq-10 (Uq-10 and UqH-10) existed in the reduced form, UqH-10. However, UqH-10 was partly oxidized during HPLC sample preparation. Therefore, the total amount of UqH-10 was taken to be the sum of UqH-10 and Uq-10. Plasma concentrations versus time plots from rats after oral administration of Uq-10, UqH-4-DMG, or UqH-DMG are shown in Figure 4A. The pharmacokinetic parameters for UqH-10 were determined by moment analysis and summarized in Table 2. The C_max_, T_max_, AUC, and MRT parameters were standardized based on control levels of UqH-10 at each time point that were individually obtained from control rats. Plasma UqH-10 levels after the administration of UqH-4-DMG and UqH-DMG were significantly elevated and reached C_max_ at 2 and 3 h, respectively. These results demonstrate that UqH-derivatives reverted to the parent form (UqH-10) in rats and acted as UqH-10-prodrugs. In contrast, plasma UqH-10 levels after Uq-10 administration remained low at approximately 0.2 μM. AUC_0–24h_ values of UqH-10 after UqH-4-DMG and UqH-DMG administration were 3.30- and 2.36-fold higher than that after Uq-10 administration, respectively. These results indicate that UqH-prodrugs can be solubilized by low bile acid levels and efficiently absorbed in rat intestines.

To determine the effects of bile secretion on the intestinal absorption of UqH-prodrugs, we established a postprandial state model to enrich bile secretion in rats. The rats were fed for only 2 h each day during the three weeks prior to administration of UqH-prodrugs and Uq-10. Plasma levels of UqH-10 after single oral administration of UqH-prodrugs and Uq-10 in the postprandial rats were significantly higher than those in fasted rats (Figure 4A,B). Even after the T_max_, plasma levels of UqH-10 in postprandial rats were maintained around 2 μM. These results suggest that abundant bile secretion induced by controlled feeding strongly promotes the solubilization of UqH-prodrugs and Uq-10. Although there are little differences in the AUC_0-24h_ values of UqH-10 between Uq-10 and UqH-prodrugs administrations under abundant bile secretion conditions, the UqH-prodrugs brought forward the T_max_ of plasma UqH to an hour earlier than after Uq-10 administration. The change in T_max_ suggests that UqH-prodrugs could solubilize into mixed-micelles upon encounter of bile acid anions, and did so more efficiently than Uq-10. The results from fasted and postprandial rats show that Uq-10 is susceptible to intestinal bile levels but UqH-prodrugs are not.

An innovative self-emulsified drug delivery system (SEDDS) for Uq-10 was previously proposed by Onoue et al [9]. They reported that an SEDDS formulation of Uq-10 improved the oral bioavailability of Uq-10 compared to crystalline Uq-10. However, this formulation technique tended to produce larger dosage volumes because it required an equal amount of fatty acid triglycerides and 8-fold more surfactant relative to Uq-10. In contrast, the current prodrug strategy for UqH-10 can be used to formulate smaller dosage volumes as they form very small particles (~5 nm) with endogenous bile acid. This is an improved procedure for developing suitable dosage formulations for easier oral uptake and enhanced intestinal diffusion.

In conclusion, UqH-4-DMG and UqH-DMG may be good prodrug candidates with enhanced intestinal absorption due to their ability to form mixed-micelles with bile acid anions. Additional screening tests to identify the best formulation of UqH-prodrugs, and additional pharmacokinetic studies will help inform future development of this important biomolecule for health and medical applications.

## 3. Materials and Methods

### 3.1. Chemicals

Uq-10 was a generous gift from Kaneka Corporation (Osaka, Japan) and was used as received. *N*,*N*-Dimethyl glycine hydrochloride was from Tokyo Kasei Kogyo Co., Ltd. (Tokyo, Japan). Eserine (physostigmine sulfate) was from Sigma-Aldrich Chemical Co. (St. Louis, MO, USA). All other chemicals were from FUJIFILM Wako Pure Chemical Corporation (Osaka, Japan). 

### 3.2. Animals

Male Sprague–Dawley (SD) rats (eight weeks old), SD rat liver microsomes, and human liver microsomes were obtained from Charles River, Japan, Inc. (Kanagawa, Japan). All animal care and use procedures were performed in compliance with the regulations established by the Experimental Animal Care and Use Committee of Fukuoka University, which are in accordance with the universal principles of laboratory animal care.

### 3.3. Instrumental Analyses

All melting points were determined with a BY-1 micro melting point apparatus (Yazawa, Tokyo, Japan) and were uncorrected. Elementary analyses, ^1^H-NMR and mass spectra measurements were performed at the Central Microanalytical Department of Pharmaceutical Sciences, Fukuoka University. The ^1^H-NMR spectra were determined at 500 MHz in CDCl_3_ using a JEOL GX-500b spectrometer (JEOL Ltd, Tokyo, Japan). The chemical shifts are expressed in δ (ppm), using tetramethylsilane as the internal standard, with the following abbreviations: s = singlet, d = doublet, m = multiplet. The coupling constant J was measured in Hz. fast atom bombardment mass (FAB-MS) spectra were obtained using a JEOL DX-300 spectrometer (JEOL Ltd, Tokyo, Japan). The ^1^H-NMR and MS spectra of UqH-derivatives are shown in the Supplemental Figures S2 and S3, respectively.

### 3.4. Synthesis of Ubiquinol-10 N,N-Dimethylglycinate Derivatives

Uq-10 (3.475 mmol) was dissolved in 150 mL distilled isopropyl ether. Sodium tetrahydroborate (9.91 mmol), suspended in methanol, was added to this ether solution and stirred for 3 min in the dark. After reduction of Uq-10 was completed, the mixture was treated with 100 mL degassed water and extracted with distilled isopropyl ether. The organic layer was dried over anhydrous sodium sulfate and evaporated in vacuo. The resulting residue, a milk-white solid, was used in the following steps as UqH-10.

*N,N*-Dimethylglycine (10.424 mmol) and dicyclohexylcarbodiimide (DCC, 10.424 mmol) in 20 mL of dry pyridine were stirred for 20 min and then UqH-10 (3.475 mmol) was added. The reaction mixture was stirred for 14 h at room temperature in the dark. 

After the reaction, the dicyclohexylurea formed was removed by filtration, and the filtrate was evaporated in vacuo. The residue was suspended in 100 mL distilled water and alkalinized with sodium bicarbonate. Dicyclohexylurea was completely removed by filtration and the filtrate was treated with 150 mL water and made alkaline with sodium bicarbonate. Then the solution was extracted with ethyl acetate. The organic layer was dried over anhydrous sodium sulfate and evaporated. The residue was fractionated on Flash 40+M silica gel columns (φ 40 × 150 mm, Biotage Japan Co., Ltd., Tokyo, Japan), eluted with a gradient of 1:1 to 2:8 (*v*/*v*) n-hexane/ethyl acetate. This yielded two fractions, a mixture of monoesters (R_f_ = 0.45) and the 1,4-bis ester (R_f_ = 0.36). *n*-Hexane containing 3N dioxane hydrochloride was added to the 1,4-bis ester fraction and the precipitate was collected and recrystallized from acetone to give the hydrochloride salt of 2,3-dimethoxy-5-methyl-6-decaprenyl-benzene-1,4-bis-*N,N*–dimethyl glycinate (UqH-DMG). The compound was a white solid.

UqH-DMG: Yield 42%. mp 179–181 °C. ^1^H-NMR (500 MHz, CDCl_3_) δ: Uq-10 moiety; 1.55–1.62 (CH_3_ × 9, m), 1.68 (CH_3_, d, *J* = 1.0), 1.72 (CH_3_, d, *J* = 1.0), 1.94–2.16 (CH_2_ × 18, m), 2.08 (CH_3_, s), 3.22 (CH_2_, d, *J* = 6.5), 3.84 (CH_3_ × 2, s), 4.88–5.13 (CH × 10, m), *N,N*-dimethylglycine moiety; 3.07 (CH_3_ × 2, s), 3.10 (CH_3_ × 2, s), 4.21 (CH_2_, s), 4.31 (CH_2_, s). FAB-MS (*m*/*z*); 1035 (M^+^-2HCl). Anal. Calcd for C_67_H_108_N_2_O_6_Cl_2_: C, 72.60; H, 9.82; N, 2.53. Found: C, 72.36; H, 9.94; N, 2.42. 

The mixture of monoesters was fractionated by HPLC. A normal phase column Mightysil Si60 (φ 20 × 250 mm, Kanto Chemical, Tokyo, Japan) and a mobile phase of n-hexane-ethyl acetate (7:3, *v*/*v*) at a flow rate of 15 mL/min, was used. The eluent was detected spectrophotometrically at 280 nm. The retention times for the 1-monoester and 4-monoester were 25.7 and 33.0 min, respectively. Corresponding fractions were collected in n-hexane containing 3N dioxane hydrochloride and isolated as the hydrochloride salts of the 1-monoester (UqH-1-DMG) and 4-monoester (UqH-4-DMG). Both were white solids. The structures of UqH-1-DMG and UqH-4-DMG were determined by using ^1^H-^13^C HMBC NMR techniques. 

UqH-1-DMG: Yield 19%. mp 82–85 °C. ^1^H-NMR (500 MHz, CDCl_3_) δ: Uq-10 moiety; 1.57–1.62 (CH_3_ × 9, m), 1.67 (CH_3_, d, *J* = 1.0), 1.71 (CH_3_, d, *J* = 1.0), 1.57–1.62 (CH_2_ × 18, m), 2.14 (CH_3_, s), 3.17 (CH_2_, d, *J* = 7.0), 3.82 (CH_3_, s), 3.90 (CH_3_, s), 4.90–5.12 (CH × 10, m), *N,N*-dimethylglycine moiety; 3.05 (CH_3_ × 2, s), 4.10 (CH_2_, s). FAB-MS (*m*/*z*); 950 (M^+^-HCl). Anal. Calcd for C_63_H_100_NO_5_Cl + 0.5H_2_O: C, 75.98; H, 10.22; N, 1.41. Found: C, 75.89; H, 10.30; N, 1.39.

UqH-4-DMG: Yield 14%. mp 75–78 °C. ^1^H-NMR (500 MHz, CDCl_3_) δ: Uq-10 moiety; 1.58–1.60 (CH_3_ × 9, m), 1.68 (CH_3_, d, *J* = 1.0), 1.76 (CH_3_, d, *J* = 1.0), 1.98–2.09 (CH_2_ × 18, m), 2.03 (CH_3_, s), 3.35 (CH_2_, d, *J* = 7.0), 3.82 (CH_3_, s), 3.90 (CH_3_, s), 5.07–5.13 (CH × 10, m), *N,N*-dimethylglycine moiety; 3.08 (CH_3_ × 2, s), 4.17 (CH_2_, s). FAB-MS (*m*/*z*); 950 (M^+^-HCl). Anal. Calcd for C_63_H_100_NO_5_Cl + H_2_O: C, 75.30; H, 10.23; N, 1.39. Found: C, 75.30; H, 10.47; N, 1.39.

### 3.5. Water Solubility

The aqueous solubility of each ester was determined by adding 20 μmol of each compound to 1 mL distilled water in amber test tubes and incubating the tubes at 25 ± 1 °C in a constant-temperature water bath. The test tubes were shaken for 24 h and the suspensions were centrifuged at 1250× *g* for 10 min. The supernatants were diluted 10-fold with distilled water and assayed by the HPLC method described in Section 3.9.1. 

### 3.6. Micellization of UqH-DMG in Water

UqH-DMG was diluted to intended concentrations with milliQ water in glass tubes. The test tubes were incubated in a water bath equipped with a thermometer. The solution appearance was visually inspected and the solubilizing points were plotted on a concentration versus incubation temperature curve.

### 3.7. Mixed-Micellization of UqH-DMG with Taurocholic Acid

#### 3.7.1. Preparation of Aqueous Solutions of UqH-DMG with Taurocholic Acid

A solution of 20 mM UqH-DMG was prepared in distilled water and incubated at 36.5 °C until the solution was translucent. The solution was combined with 10 mM TCA aqueous solution in the final molar ratios of 1:0.5–10 UqH-DMG:TCA.

#### 3.7.2. Determination of Particle Sizes

The Z-average of diameters of the particles of aqueous solution of UqH-DMG prepared in a polystyrene cuvette in Section 3.7.1. above were determined by a Zetasizer Nano ZS (MALVERN, Worcestershire, UK). The measurements were performed three independent times for each sample.

### 3.8. Enzymatic Hydrolysis of UqH-Derivatives

The hydrolysis of esters was analyzed at 37 °C in phosphate buffered saline (PBS) containing commercially available rat or human liver microsomes (In Vitro Technologies, Inc., Baltimore, MD, USA). The microsomes (20 mg/mL) were adjusted to a protein concentration of 2 mg/mL and were preincubated at 37 °C for 5 min before adding the esters. 

Stock solutions of esters were prepared in ethanol. The enzymatic reactions were initiated by adding 10 µL ester stock solution (final concentration 0.1–0.4 mM) and 50 μL PBS to 940 µL of preheated reaction medium containing rat or human liver microsomes in amber test tubes. These reactions were incubated at 37 °C and, at various times, 100 μL aliquots were removed and mixed with 100 µL 10% trichloroacetic acid. Samples were then vortex mixed for 2 min and 800 µL ethyl acetate was added, with vortex mixing for 5 min. The samples were centrifuged at 1250× *g* for 10 min. The 20 µL organic layer was analyzed by the HPLC system described in Section 3.9.1. The initial hydrolytic rate (mM UqH-10 formed per min) was calculated from the initial slope of a plot of UqH-10 concentration versus time. 

Effects of eserine on UqH-4-DMG hydrolysis in liver microsomes was also examined, using similar methods, with 50 µL eserine aqueous solution added, instead of PBS, to the mixtures with liver microsomes. Eserine was tested at 0–2.0 mM.

### 3.9. HPLC Analysis

#### 3.9.1. HPLC System and Conditions for Water Solubility and Hydrolysis Study

The Shimadzu HPLC system (Shimadzu, Kyoto, Japan) consisted of a pump (LC-10AD), an autoinjector (SIL-10ADvp), a UV detector (SPD-10Avp), and a column oven (CTO-10Avp). The eluent was monitored spectrophotometrically at 280 nm. Compounds were analyzed on a YMC-Pack C8 reversed-phase column (φ 4.6 × 250 mm, YMC Co., Ltd., Kyoto, Japan) protected by a µBondapak C18 Guard-Pak (Waters Co., Milford, MA, USA) with a degassed mobile phase of methanol and 2-propanol (850/150, *v*/*v*) at 0.8 mL/min. UqH-10 and Uq-10 were quantified using linear calibration curves of peak areas versus concentrations. UqH-10 was partly oxidized during the preparation procedure. Therefore, the sum of UqH-10 and Uq-10 represented total UqH-10. Retention times were: UqH-10, 11.6 min; UqH-ester derivatives, 13.2 min and Uq-10, 15.4 min.

#### 3.9.2. HPLC System and Conditions for Pharmacokinetic Study

The HPLC system consisted of a pump (Model LC-10AD, Shimadzu), an autoinjector (Model SIL-10ADvp, Shimadzu) and a column oven (Model CTO-10Avp, Shimadzu). Compounds were analyzed using a CAPCELL PAK C18 column (φ 4.6 × 150 mm, particle size 3 μm, Shiseido, Tokyo, Japan), protected by a Nova-Pak C18 (φ 3.9 × 20 mm, particle size 4 μm, Waters Co.) guard column, with a mobile phase consisting of 0.7% sodium perchlorate monohydrate in methanol/ethanol/70% perchloric acid (400/600/1, *v*/*v*/*v*) delivered at flow rate 0.6 mL/min. The peak area was used to quantify UqH-10. 

The detection system consisted of a Coulochem II electrochemical detector (ESA, Inc., Chelmsford, MA, USA), connected with two cells. After separation, Uq-10 was reduced to UqH-10 by passing through the Model 5020 guard cell (–450 mV) and was oxidized by passing through the Model 5011 analytical cell (electrode 1, –100 mV; electrode 2, +350 mV). The signals of electrode 2 were transmitted to the detector (filter, 10 sec; output, 1 V; offset, 20%; gain range, 0–7 min = 500 nA, 7–22 min = 10 nA). Chromatographic data were integrated using CLASS-vp (Shimadzu, Japan). UqH-10 and Uq-10 were quantified using linear calibration curves of peak areas versus compound concentrations. Retention times were: UqH-DMG, 4.8 min; UqH-1-DMG, 6.6 min; UqH-4-DMG, 6.7 min; UqH-10, 11.4 min and Uq-10, 16.8 min.

### 3.10. Dosing Protocol

We first performed a preliminary experiment to determine the effects of food intake on gastrointestinal absorption of Uq-10 in rats. The contents of commercially available hard capsules (Neuquinon^®^ capsules, Eisai Co., Ltd., Tokyo, Japan) were used as the source of Uq-10. The Uq-10 preparation was suspended in distilled water containing 20% (*w*/*v*) arabic gum. Before the experiment, the fasting group was fasted for 16 h prior to dosing. In the controlled-feeding group, feeding for 2 h per day was continued for 3 weeks before dosing and Uq-10 was administered 2 h after the feeding time. All rats were supplied with drinking water ad libitum. Uq-10 was administered via the esophagus with a gastric tube. Rats were administered 40.5 mmol/kg Uq-10 in 0.2 mL/100 g body weight and were given no food after administration. At 0.5, 1, 2, 3, 4, 6, 8, and 24 h, blood samples (200 µL) were collected from the external jugular vein under anesthesia with isoflurane using heparinized syringes. UqH-10 and Uq-10 were extracted as described by Yamashita and Yamamoto [15]. The plasma was isolated by centrifugation, and 50 µL plasma aliquots were added to 250 µL methanol. After vortex mixing for 30 s, 500 µL n-hexane was added, followed by vortex mixing for 2 min. The samples were diluted with ethyl acetate and centrifuged at 1250× *g* for 3 min. A 5 µL aliquot of each organic layer was analyzed by the HPLC system described in Section 3.9.2. 

Based on results of this initial experiment, we next treated rats with Uq-10, UqH-4-DMG and UqH-DMG, using the same procedures. UqH-4-DMG and UqH-DMG were suspended in a 20% propylene glycol aqueous solution. All compounds were administered at 40.5 mmol/kg.

## Figures and Tables

**Table 1:** Kinetic parameters for hydrolysis of UqH-DMG, UqH-1-DMG and UqH-4-DMG in rat and human liver microsomes at pH 7.4 and 37 °C.

Parameters	UqH-DMG ^a^	UqH-1-DMG	UqH-4-DMG	
	Rat			Human
*K*_m_ (×10^−3^ M)	3.89 ^a^	4.63	0.0751	0.0722
*V*_max_ (×10^−6^ M·min^−1^)	0.603 ^a^	0.821	0.119	0.123
*V*_max_*/K*_m_ (×10^−3^ min^−1^)	0.155 ^a^	0.177	1.58	1.70

The values are obtained from Michaelis–Menten curve fitting (GraphPad Prism). ^a^ The kinetic parameters were calculated using the generated UqH-10 levels after a sequential hydrolysis process.

**Table 2:** Pharmacokinetic parameters for UqH-10 after oral administration of UqH-DMG, UqH-4-DMG, or Uq-10 in fasted and postprandial rats.

Parameters	Uq-10	UqH-4-DMG	UqH-DMG	Uq-10	UqH-4-DMG	UqH-DMG
	Fasted			Postprandial		
*n*	4	5	6	3	3	3
Dose (μmol·kg^−1^)	40.5					
*C*_max_ (μmol·L^−1^)	0.143 ± 0.0826	0.763 ± 0.209	0.800 ± 0.157	1.99 ± 0.472	3.01 ± 0.423	2.24 ± 0.110
*T*_max_ (h)	1	2	3	4	3	3
*AUC*_0-24h_ (μmol·L^−1^·h)	2.16 ± 0.878	7.13 ± 1.71	5.10 ± 1.57	35.0 ± 1.78	39.3 ± 6.78	34.4 ± 6.51
*MRT* (h)	8.56 ± 1.96	8.96 ± 0.710	8.43 ± 0.956	12.2 ± 0.971	9.12 ± 0.565	11.5 ± 0.525

Values are each the mean ± SE. The calculated parameters, C_max_, T_max_, AUC and MRT, were standardized by base levels of plasma UqH-10 at each point obtained from control rats.

## Supplementary Materials

The following are available online, Figure S1. Effects of eserine on enzymatic hydrolysis of UqH-4-DMG by rat and human liver microsomes.
