# Supplementary material for: Novel Cationic Prodrug of Ubiquinol-10 Enhances Intestinal Absorption via Efficient Formation of Nanosized Mixed-Micelles with Bile Acid Anions
Source: Molecules. 2020 Jan 27;25(3):546. doi: 10.3390/molecules25030546 (PMC7037610; doi:10.3390/molecules25030546)
Supplement: Supplementary file 1 [file molecules-25-00546-s001.pdf]

Supplemental Figure S1

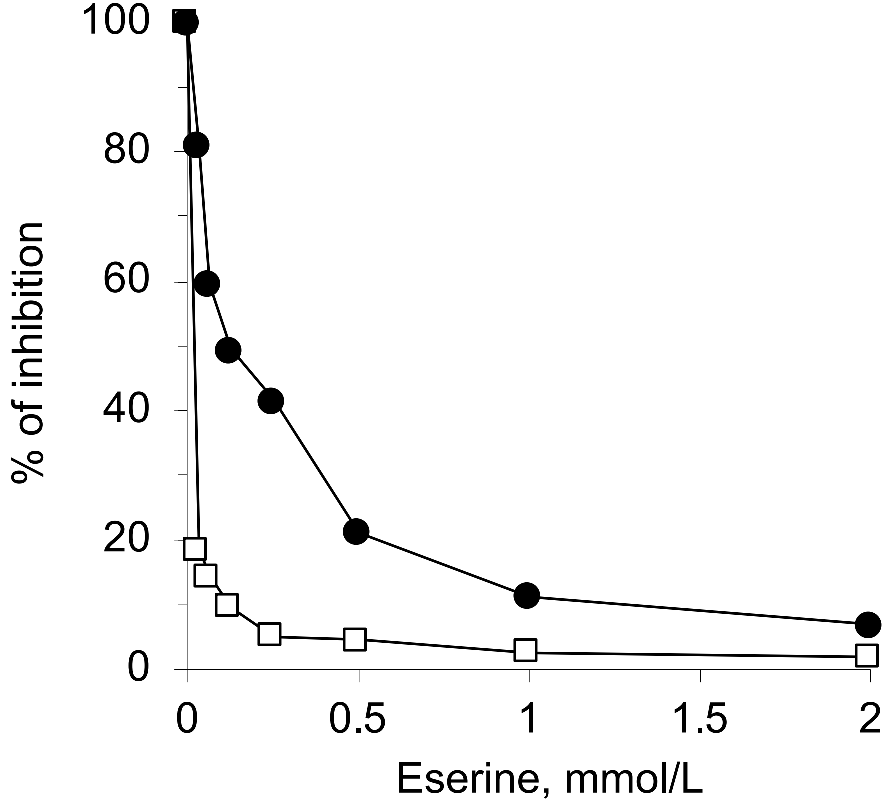

Figure S1. Effects of eserine on enzymatic hydrolysis of UqH-4-DMG by rat and human liver microsomes.
(●), rat liver microsomes; (□), human liver microsomes.

Supplemental Figure S2

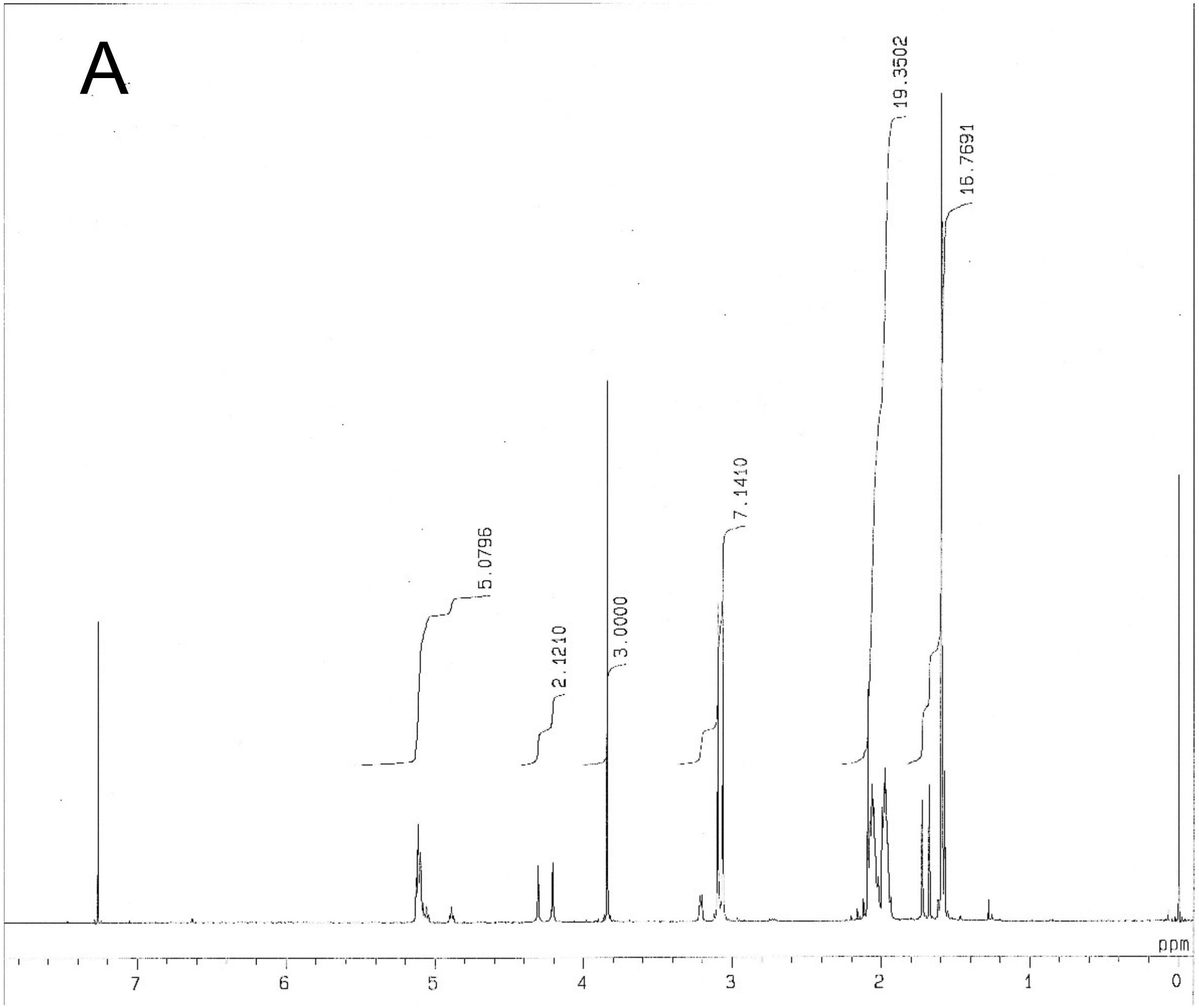

Supplemental Figure S2

B

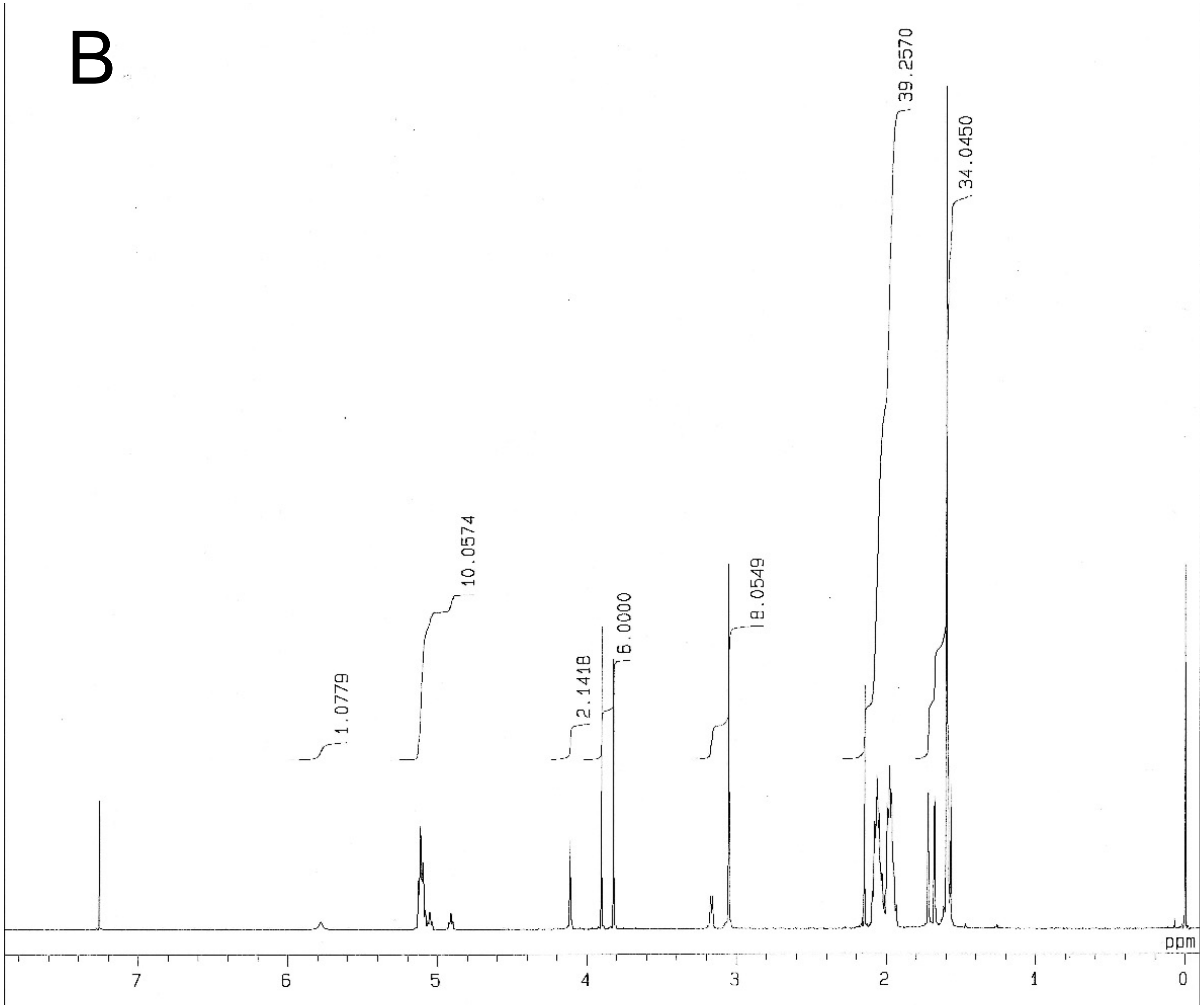

Supplemental Figure S2

C

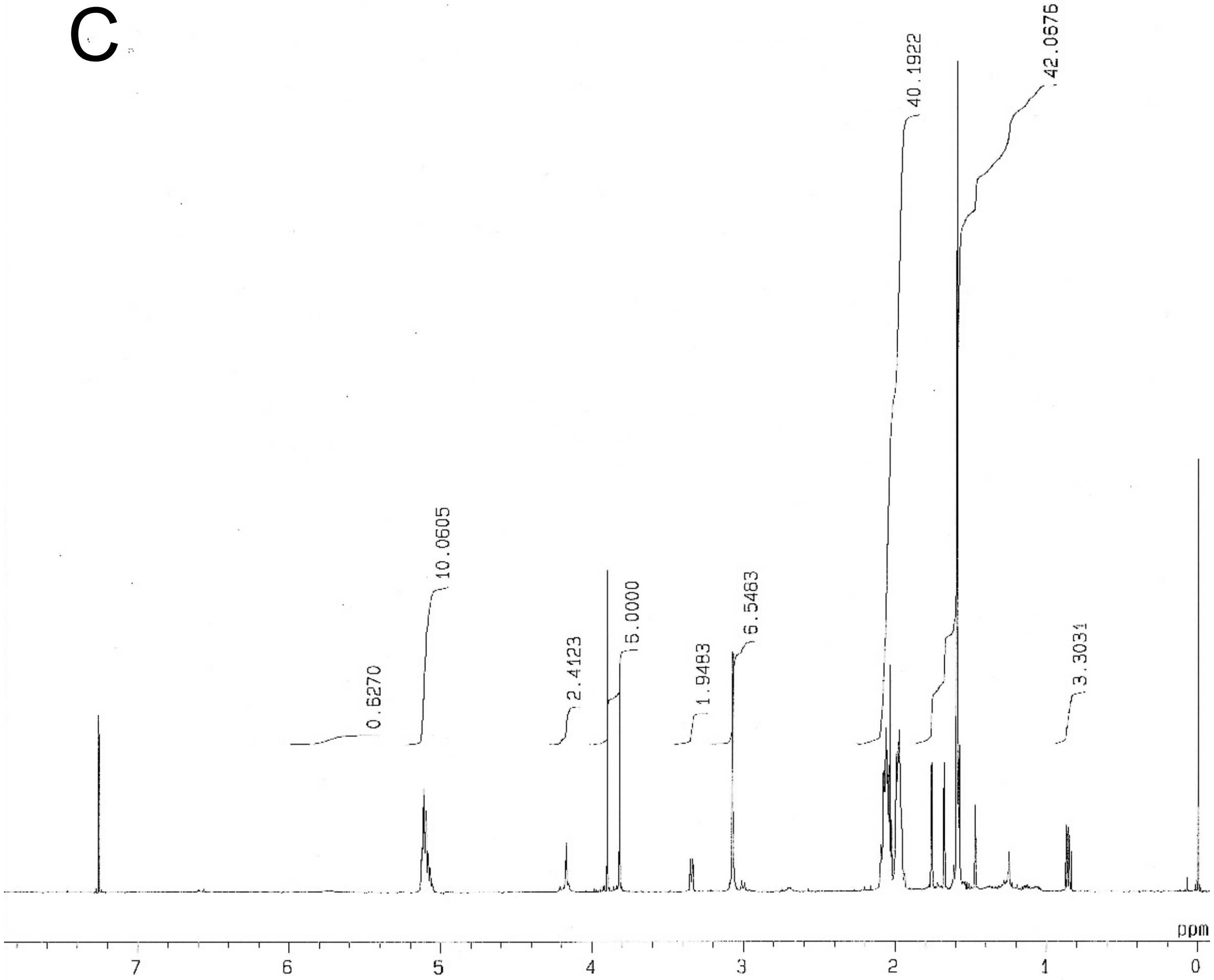

Figure S2. ^{1}H-NMR spectra of UqH-DMG (A), UqH-1-DMG (B) and UqH-4-DMG (C).

Supplemental Figure S3

A

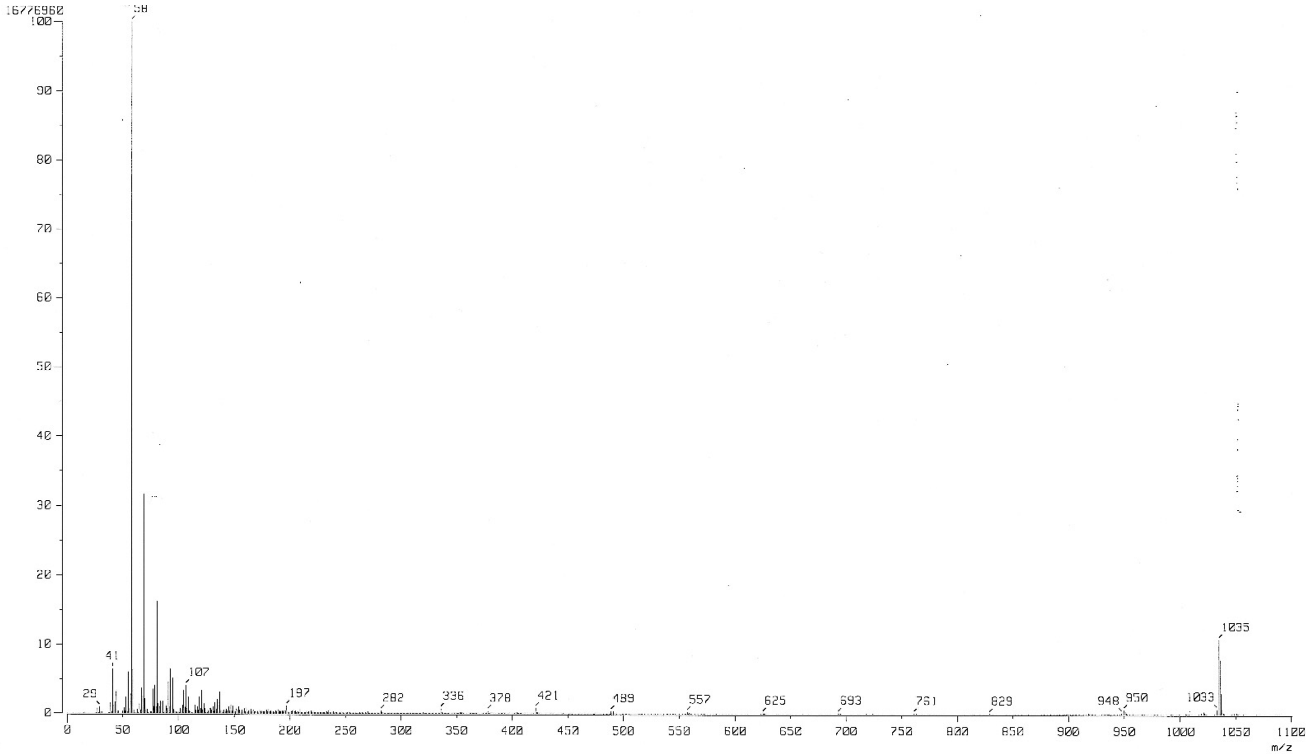

Supplemental Figure S3

B

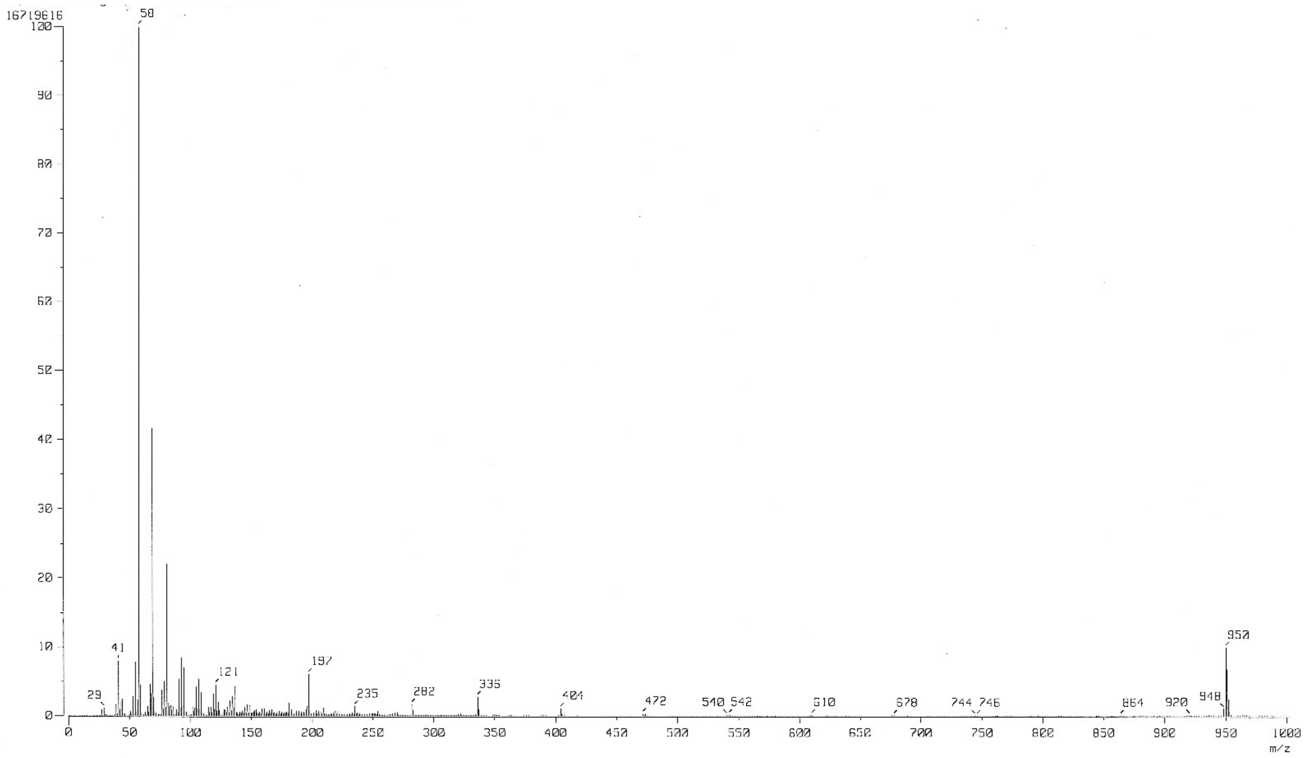

Supplemental Figure S3

C

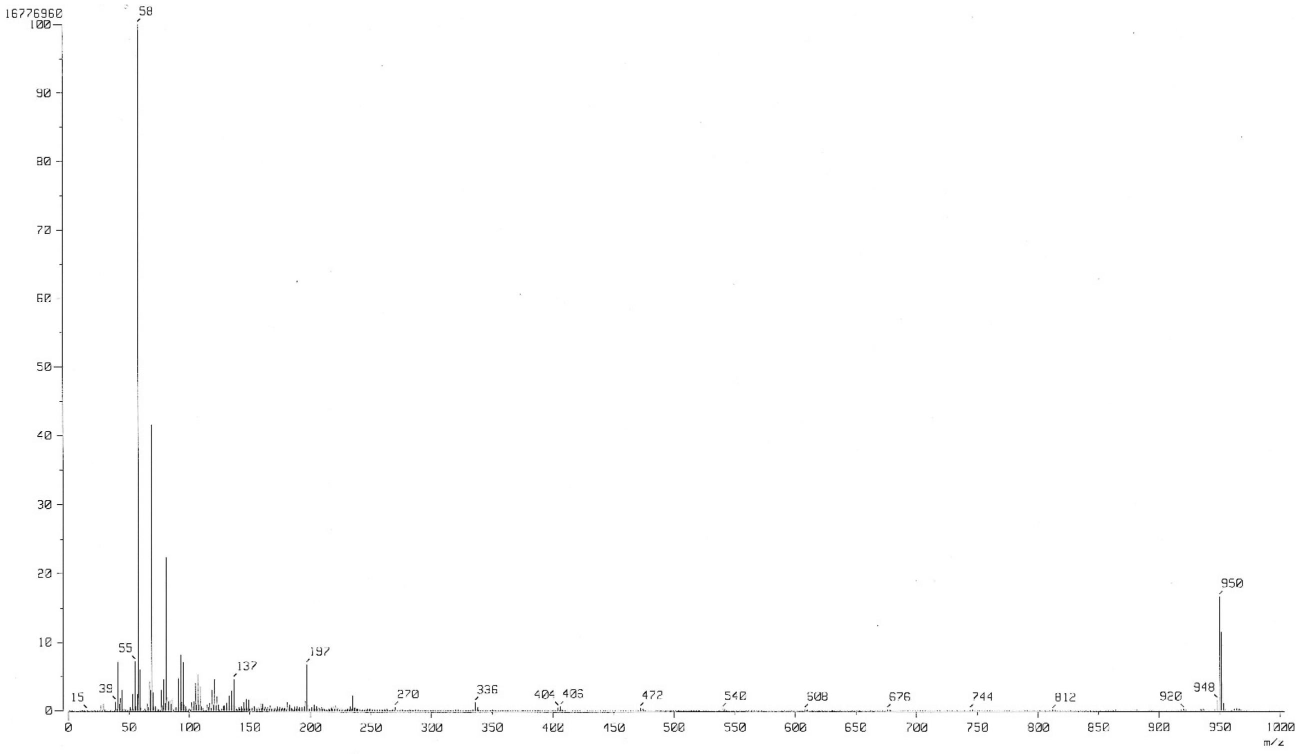

Figure S3. MS spectra of UqH-DMG (A), UqH-1-DMG (B) and UqH-4-DMG (C).
